# Experimental and Modeling Analyses of Human Motion Across the Static Magnetic Field of an MRI Scanner

**DOI:** 10.3389/fbioe.2021.613616

**Published:** 2021-05-05

**Authors:** Davide Gurrera, Alberto Leardini, Maurizio Ortolani, Stefano Durante, Vittorio Caputo, Karmenos K. Gallias, Boris F. Abbate, Calogero Rinaldi, Giuseppina Iacoviello, Giuseppe Acri, Giuseppe Vermiglio, Maurizio Marrale

**Affiliations:** ^1^Advanced Radiation Oncology Department, Cancer Care Center, Istituto di Ricovero e Cura a Carattere Scientifico (IRCCS) Sacro Cuore Don Calabria Hospital, Negrar di Valpolicella, Italy; ^2^Dipartimento di Fisica e Chimica, Università degli Studi di Palermo, Palermo, Italy; ^3^Istituto di Ricovero e Cura a Carattere Scientifico (IRCCS) Istituto Ortopedico Rizzoli, Movement Analysis Laboratory, Bologna, Italy; ^4^Azienda Ospedaliera di Rilievo Nazionale e di Alta Specializzazione (A.R.N.A.S.) Civico–Di Cristina–Benfratelli, Unità Operativa Complessa (U.O.C.) Fisica Sanitaria, Palermo, Italy; ^5^Villa Santa Teresa, Unità Operativa (U.O.) Fisica Sanitaria, Bagheria, Italy; ^6^Dipartimento di Scienze Biomediche, Odontoiatriche e delle Immagini Morfologiche e Funzionali (BIOMORF), Università degli Studi di Messina, Messina, Italy; ^7^Scuola di Specializzazione in Fisica Medica, Università degli Studi di Messina, Messina, Italy

**Keywords:** human movement analysis, static magnetic fields, exposure limit values, MRI personnel safety, Directive 2013/35/EU

## Abstract

It is established that human movements in the vicinity of a permanent static magnetic field, such as those in magnetic resonance imaging (MRI) scanners induce electric fields in the human body; this raises potential severe risks of health to radiographers and cleaners exposed routinely to these fields in MRI rooms. The relevant directives and parameters, however, are based on theoretical models, and accurate studies on the simulation of the effects based on human movement data obtained in real conditions are still lacking. Two radiographers and one cleaner, familiar with MRI room activities and these directives, were gait analyzed during the execution of routine job motor tasks at different velocities. Full body motion was recorded in a gait laboratory arranged to reproduce the workspace of a room with an MRI full-body scanner. Body segments were tracked with clusters of at least three markers, from which position and velocity of the centroids were calculated. These were used as input in an established computer physical model able to map the stray field in an MRI room. The spatial peak values of the calculated electric field induced by motion of the head and of the entire body during these tasks, for both the health and sensory effects, were found smaller than the thresholds recommended by the European directives, for both 1.5 T and 3.0 T MRI. These tasks therefore seem to guarantee the safety of MRI room operators according to current professional good practice for exposure risks. Physical modeling and experimental measures of human motion can also support occupational medicine.

## 1. Introduction

Magnetic resonance imaging (MRI) is used largely worldwide to assess the status of tissues in patients with musculoskeletal and other diseases. There are ~50,000 MRI machines worldwide, with about 5,000 new units sold every year. Forty million scans are performed annually in the United States, and the number of examinations in 2017 has the peak of 143 per 1,000 population in Germany (Mikulic, 2019). It is also established that movements in the vicinity of a permanent static magnetic field, such as that of an MRI scanner, induce electric fields in the human body. This raises potential severe risks of health to the persons working routinely in MRI rooms, such as radiographers and cleaners. There are directives for these workers on how to move in these rooms, but today these are based on theoretical models only (inter alii, Hartwig et al., 2014, 2019; Zilberti et al., 2015, 2016; Sannino et al., 2017). In other words, accurate simulation studies based on human movement data obtained in real conditions able to provide estimations of the exact effects of these fields are still lacking. The scope of the present experimental and modeling study is to provide these estimations for the first time by enhancing with experimental data the analysis provided in a previous modeling work (Gurrera et al., 2019). The general aim is to support general good practice methods for exposure assessment of personnel moving across the permanent static magnetic field straying from MRI scanners.

This previous work in fact (Gurrera et al., 2019) suffers from some limitations. The MRI operators were supposed to move translationally and at a constant speed, with relevant parameters derived on the basis of general studies reporting allegedly normal walking speeds (Bohannon and Williams Andrews, 2011). Thus, there is still a lack of specialized literature or experiments dealing with motion analysis of MRI operators. Moreover, the exposure assessment did not include any 3.0 T facilities. What would be necessary for the enhancement of the previous work is a full body, i.e., from head to foot, kinematic characterization of real MRI operators at work. This would provide the most realistic estimation of the electric field induced by these movements, easily extendable to the scanners of 3.0 T or more.

This kinematic characterization is obtained usually by human movement analysis (HMA) using stereophotogrammetry, an established technique which allows for accurate 3D tracking in space of body segments during the execution of locomotion tasks or elementary exercises or even high-performance sport activities (Cappozzo et al., 2005). This is achieved by instrumenting the subject under analysis with small spherical markers stuck on the skin possibly according to standard protocols (Ferrari et al., 2008; Kainz et al., 2017), and in case by arranging the gait analysis laboratory with the necessary furnishing for the simulation of the required environment according to the activity under investigation (chairs, steps, stairs, obstacles, etc.).

As mentioned, given an inertial reference frame in which the magnet is at rest, electric charges movements within the body of an MRI operator may occur because of body segment voluntary movements of the subjects; other organ and tissue motion within the body (Herman, 2016) are here ignored. The motion-induced field within the body of an MRI operator is addressed in the guidelines of the International Commission on Non-Ionizing Radiation Protection (ICNIRP) (International Commission on Non-Ionizing Radiation Protection, 2014). Unfortunately, for these estimations, questionable approximations were used, and an alleged “conversion factor” should be accounted, associated to “the location within the body, the size of the body, the shape of the body, electrical properties of the tissue” as well as “the direction and distribution of the magnetic field.” Direct measurements of body segment motion of MRI operators are therefore necessary to quantify these effects.

The present work wants to contribute in this respect. In particular, the hypothesis is that the exposure of MRI personnel to static magnetic fields does comply with the current European Directive 2013/35/EU, and for both 1.5 T and 3.0 T MRI machines.

## 2. Materials and Methods

### 2.1. The Theoretical Basis

The theoretical basis, introduced and discussed in Gurrera et al. (2019), is here briefly summarized.

1. Given an inertial reference frame in which the magnet is at rest, electric charges within the body of an MRI operator may be moving because of two reasons: natural movements within the body as those related to blood flow and nervous communication system (Herman, 2016), which are ignored in the present study, and the voluntary movements of the body segments and joints associated to locomotion and upper body maneuvers according to the tasks to be performed.
2. Let γ(*t*) be an oriented closed conducting wire moving across a static magnetic field ${\vec{B}}_{\text{ext}}$, $\vec{v}$ the velocity of each point of the wire, *S*_γ(*t*)_ an arbitrary surface enclosed by γ(*t*).
  Then, according to the Faraday–Neumann's law:
  (1) $$\begin{array}{l} \oint_{\gamma (t)}\vec{E}\cdot \hat{t}dl=-\iint_{S_{\gamma (t)}}\frac{\partial \vec{B}}{\partial t}\cdot \hat{n}dS \end{array}$$
  or equivalently:
  (2) $$\begin{array}{l} \oint_{\gamma (t)}\left(\vec{E}+\vec{v}\times \vec{B}\right)\cdot \hat{t}dl=-\frac{d}{dt}\iint_{S_{\gamma (t)}}\vec{B}\cdot \hat{n}dS \end{array}$$
  where $\vec{E}$ and $\vec{B}$ are the electric field and the overall magnetic field, respectively. The rest of the notation is assumed to be familiar to the reader.
  If the inductance of the wire is negligible, then it is possible to assume that $\vec{B}={\vec{B}}_{\text{ext}}$ and there is no time dependence. Therefore, according to Equation (1), the generated electric field comes out to be irrotational and, as a consequence, has to be caused only by a continuous charge redistribution within the wire. And such a redistribution is imposed by the action of the Lorentz' force. The complete cause and effect picture is as follows. Free charges within the conductor start moving across the external magnetic field with a mean velocity that, at each point, is equal to the velocity of the wire. Then the Lorentz' force accelerates them and causes a charge separation that produces an electrostatic field $\vec{E}$ opposing further accumulation, i.e., opposing the electromotive field $\vec{v}\times \vec{B}$. Except for particular given arrangements of ${\vec{B}}_{\text{ext}}$ and $\vec{v}$, equilibrium is not reached and a time-varying density current flows along the wire. As far as the magnetic field produced by this induced current may be neglected, there will be only an electromotive field $\vec{v}\times \vec{B}$ and a reaction electrostatic field ($\nabla \times \vec{E}=\vec{0}$). Now, a human body moving inside an MRI room may be considered as a special instance of a massive extended non-ferromagnetic conductor for which all the reasoning above applies. Particularly, the motion-induced “electric field” to be tested against the exposure limit values (ELVs) of the directive will be the sum of the electromotive field $\vec{v}\times \vec{B}$ and the reaction electrostatic field $\vec{E}$. If the latter, which even a computational model accounting for the electrical properties of the human body could only approximate, is neglected, then an overestimation of the induced field may be expected. But that even results in a precautionary approach.
  To summarize, an electric charge moving in a magnetic field $\vec{B}$, with overall instantaneous velocity $\vec{v}$, is subjected to the Lorentz' force and the related electromotive field $\vec{v}\times \vec{B}$ may be regarded as the best approximation for the motion-induced field within the body of an MRI operator. Indeed, the induced field suggested in the guidelines of the International Commission on Non-Ionizing Radiation Protection (ICNIRP) (International Commission on Non-Ionizing Radiation Protection, 2014)
  (3) $$\begin{array}{r} E_{\text{i}}=C\frac{\text{d}B}{dt} \end{array}$$
  may be considered as an unnecessary coarse approximation of it (and, possibly, an overestimation), *C* being a “conversion factor” that should account for “the location within the body, the size of the body, the shape of the body, electrical properties of the tissue” as well as “the direction and distribution of the magnetic field.” In fact, in a static magnetic field, the electric field induced in a moving conducting loop is irrotational (see also Bringuier, 2002). Therefore, the Faraday–Neumann's law simplifies to:
  (4) $$\begin{array}{l} \oint \vec{v}\times \vec{B}\cdot \hat{t}\;dl=-\frac{d}{dt}\iint \vec{B}\cdot \hat{n}\;dS \end{array}$$
  and calculating the rate of change of the magnetic flux is tantamount to calculate the circulation of $\vec{v}\times \vec{B}$.
3. A magnetic dipole may provide (if data confirming evidence is obtained) a parsimonious, but still adequate, 3D approximation of the magnetic field straying from a closed full-body MRI scanner, its specific architecture details being unknown (see also Sannino et al., 2017).

### 2.2. Mapping Relevant Velocities in an MRI Room

**Figure 1:**
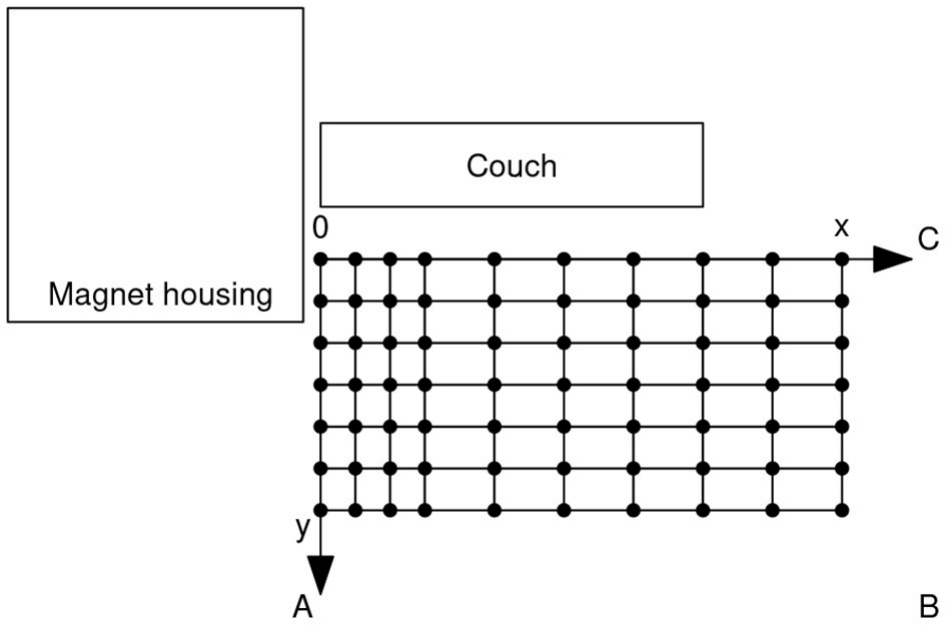
Measurement environment where the three volunteers executed the tasks listed in Table 2. In order to account for different MRI room arrangements, A–C indicate the possible position of the door. In the actual MRI room, the modulus of the stray field was measured in each of the indicated points and at each of three different heights. The short and long steps in the net are 20 and 40 cm long, respectively. The map lies as close as possible to the bore and the couch.

In May 2019, three healthy MRI operators, specific job and other details provided in Table 1, volunteered to contribute the current study and to be observed and recorded during the execution of routine job tasks at the Movement Analysis Laboratory of the Istituto Ortopedico Rizzoli in Bologna (Italy). This was instrumented with an 8-TV-camera stereophotogrammetric system (Vicon Motion Systems, Oxford, UK) and related processing software and opportunely arranged, as sketched in Figure 1, to reproduce a typical workspace of an MRI closed full-body scanner. The subjects were given instructions and time to familiarize with the room environment and then were asked to execute twice each of the representative job motor tasks, involved in normal and emergency scenarios, listed in Table 2.

**Table 1:** Specific job and other details of the three MRI operators who volunteered to participate the study.

**Operator**	**Job**	**Gender**	**Age**	**Height (cm)**	**Weight (kg)**
MRIR1	Radiographer	Male	31	189	115
MRIR2	Radiographer	Female	27	150	49
MRIC	Cleaner	Male	54	172	61

**Table 2:** Selected representative job motor tasks, involved in normal and emergency scenarios, executed by the MRI operators who volunteered to participate the study.

**Task code**	**Involved operators**	**Task title**	**Task description**
N1	Radiographers	Entering/Leaving A	The operator enters the MRI room through Door A, reaches the control panel on the magnet housing, then leaves the room through the same door.
N2	Radiographers	Entering/Leaving B	As in N1, but through Door B.
N3	Radiographers	Entering/Leaving C	As in N1, but through Door C.
N4	Radiographers	Head coil preparation	The operator enters the MRI room through Door A and places the head coil on the couch near the bore entrance, then leaves the room through the same door.
N5	Radiographers	Patient centering	The operator is near the bore entrance, bends over the patient and positions the head coil, centers and checks the patient, then leaves the room through Door A.
N6	Radiographers	Object recovering	The operator enters the MRI room through Door A, bends over the floor next to the bore entrance and picks up an object, then leaves the room through the same door.
E1	Radiographers	Emergency entering A	Patient alarm on. The operator enters the MRI room through Door A and checks the patient lying inside the bore, then leaves the room through the same door.
E2	Radiographers	Emergency entering B	As in E1, but through Door B.
E3	Radiographers	Emergency entering C	As in E1, but through Door C.
E4	Radiographers	Emergency patient extraction	Patient alarm on. The operator enters the MRI room through Door A, checks the patient lying inside the bore, then rapidly extracts the couch manually and finally leaves the room through the same door.
C1	Cleaner	Floor sweeping	The operator enters the MRI room through Door A and sweeps the floor (from the door toward the couch and then back), then leaves the room through the same door.
C2	Cleaner	Floor mopping	The operator enters the MRI room through Door A and mops the floor (from the couch toward the door), then leaves the room through the same door.

*Refer to Figure 1 for a sketch of the measurement environment. The words “MRI room,” “door,” “control panel,” “magnet housing,” “coil,” “couch,” “bore,” and “patient” are used only evocatively. However, during the acquisition the floor was really swept and mopped by the cleaner and the patient alarm was really heard during the emergency scenarios*.

State-of-the-art stereophotogrammetric HMA was performed during the execution of the tasks. Established protocols were used to track lower limbs and pelvis segments (Leardini et al., 2007), trunk and shoulders (Leardini et al., 2011), head, and upper limbs (according to Plug-in-Gait protocol, Vicon Motion Systems, Oxford, UK). All together, combining the three protocols, a total of 47 spherical reflective markers, 14 mm diameter, were stuck on the skin in correspondence of palpable anatomical landmarks, each tracked in space at 100 Hz by the stereophotogrammetric system during movement. Six of these markers served only for anatomical calibration of those landmarks necessary for body segment analysis and were removed after a single static posture acquisition in double-leg stance. Marker trajectories were smoothed by the standard software tools within the motion capture system, according to established algorithms (Woltring, 1985). Then, from each cluster of markers stuck in a single body segment, the corresponding centroid was also derived. From each marker and centroid trajectory, the corresponding velocity was computed. In particular, instantaneous position and velocity during the execution of the tasks were obtained for each of the 23 representative body points listed in Table 3.

**Table 3:** Selected points in the body of the three operators whose instantaneous position and velocity during the execution of the tasks are used for the exposure assessment.

**Point number**	**Point type**	**Point position**
1	Centroid	Head
2	Centroid	Trunk
3	Centroid	Pelvis
4	Centroid	Right thigh
5	Centroid	Left thigh
6	Centroid	Right tibia
7	Centroid	Left tibia
8	Centroid	Right foot
9	Centroid	Left foot
10	Centroid	Right hand
11	Centroid	Left hand
12	Marker	Right temple
13	Marker	Left temple
14	Marker	Right side of occipital bone
15	Marker	Left side of occipital bone
16	Marker	Second thoracic vertebra
17	Marker	Midpoint between the inferior angles of most caudal points of the two scapulae
18	Marker	Neck in between jugular veins
19	Marker	Xiphoid process
20	Marker	Right anterior superior iliac spine
21	Marker	Left anterior superior iliac spine
22	Marker	Right posterior superior iliac spine
23	Marker	Left posterior superior iliac spine

### 2.3. Mapping the Stray Field in an MRI Room

The model proposed in Gurrera et al. (2019) for the static magnetic field straying inside an MRI room was applied in the present study to a closed full-body 3.0 T scanner, the modulus of the magnetic field being mapped, in order to fit the model, as described in the paper and by the same three-axis Hall magnetometer. Particularly, the modulus of the magnetic field was measured according to the map shown in Figure 1, positioned as close as possible to the bore and the couch and whose short and long steps are 20 and 40 cm long, respectively. These measurements were recorded in each of the 70 indicated points and at each of three different heights from the floor level: 72, 119, and 156 cm. Therefore, a three-dimensional map composed of 210 measures was produced for the scanner. The modulus of the generated magnetic dipole and its height above the floor level, i.e., the preliminary estimations necessary for the fit (Gurrera et al., 2019), are 1.06 MA m2 and 100 cm, respectively.

### 2.4. Implementation

What follows was carried out in the R environment for statistical computing and visualization (R Core Team, 2018) by *ad hoc* in-house developed code.

## 3. Results

### 3.1. MRI Operators: How Fast Do They Move?

**Figure 2:**
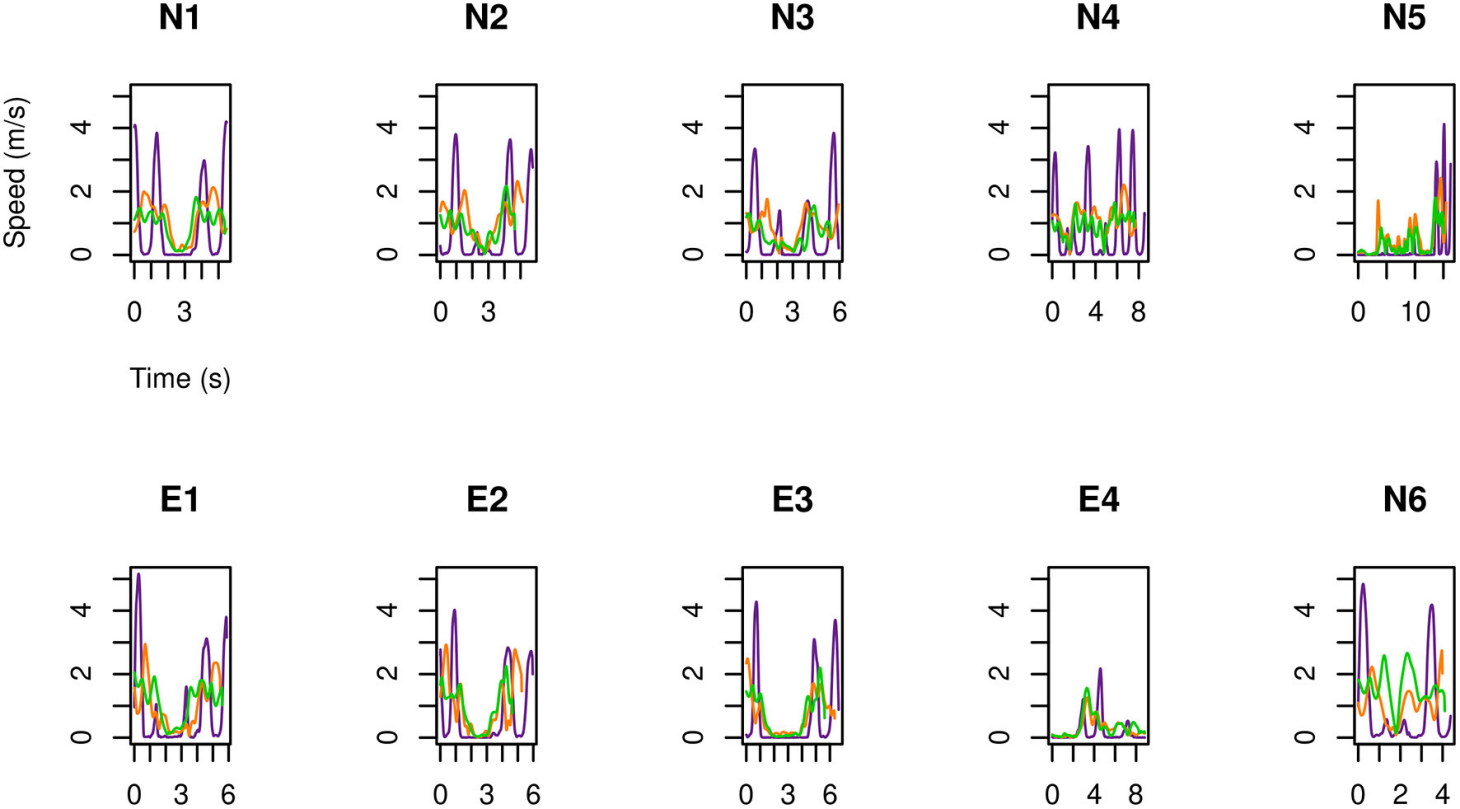
For MRIR1 (Table 1), speed of the head and of the limbs as a function of time during the execution of each of the recurrent selected tasks (Table 2). Particularly, the green line refers to the marker on the right temple, the orange line to the right hand, and the violet line to the right foot.

**Figure 3:**
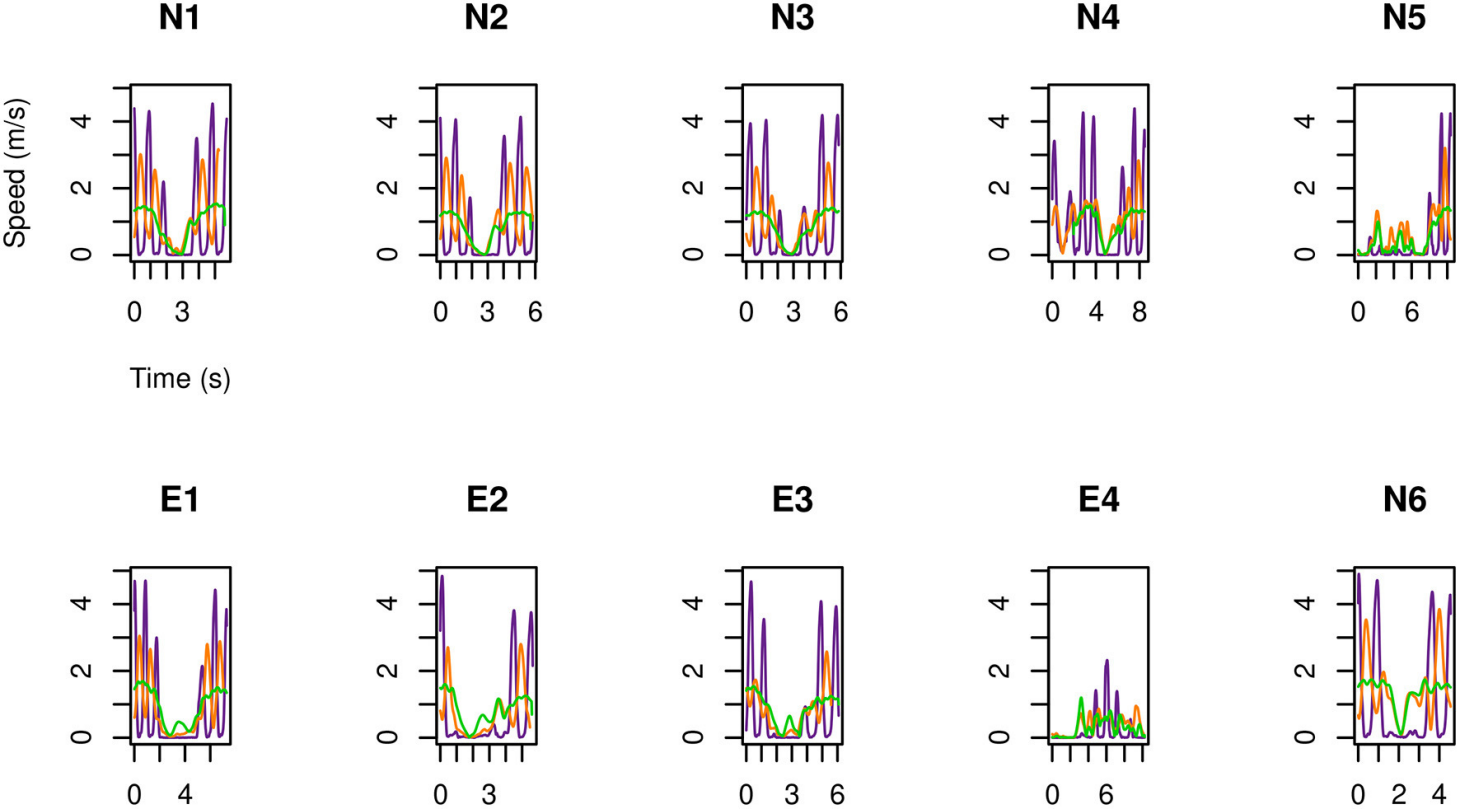
As in Figure 2, but for MRIR2.

**Figure 4:**
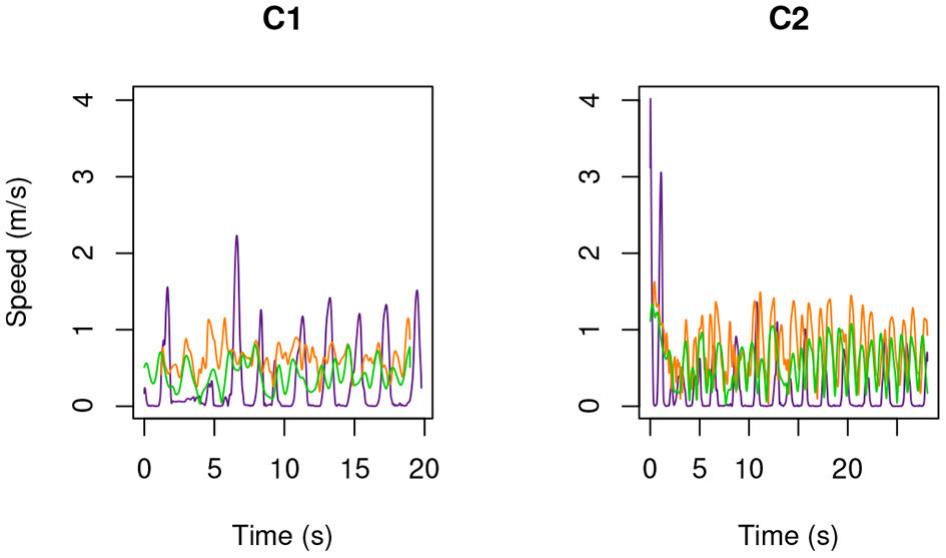
As in Figure 2, but for MRIC.

As a first result of the HMA, displayed in Figures 2–4 is the speed, as a function of time, of each of the three examined operators (Table 1) during the second execution of each of their tasks (Table 2). No significant difference was observed between the first and the second execution of each task. Specifically, position and velocity of the body segments here analyzed showed a good inter-trial repeatability, consistent with previous work from these authors (Manca et al., 2010; Caravaggi et al., 2011) where standard deviations of gait-analysis patterns are in the range 2÷4%.

The following results are drawn.

1. The feet are the part of the body that move fastest: up to 5 m s-1. Then come the hands: up to 3.5 m s-1.
2. Head marker on MRIR2 moved always slower than 2 m s-1, while this speed was occasionally exceeded by MRIR1. In the case of MRIC, head speed did not exceed 1 m s-1.
3. For the two radiographers, a U-shaped speed profile is observed: operators move fast when entering and leaving the room, while they slow down as they approach the magnet.

### 3.2. The Fit

**Figure 5:**
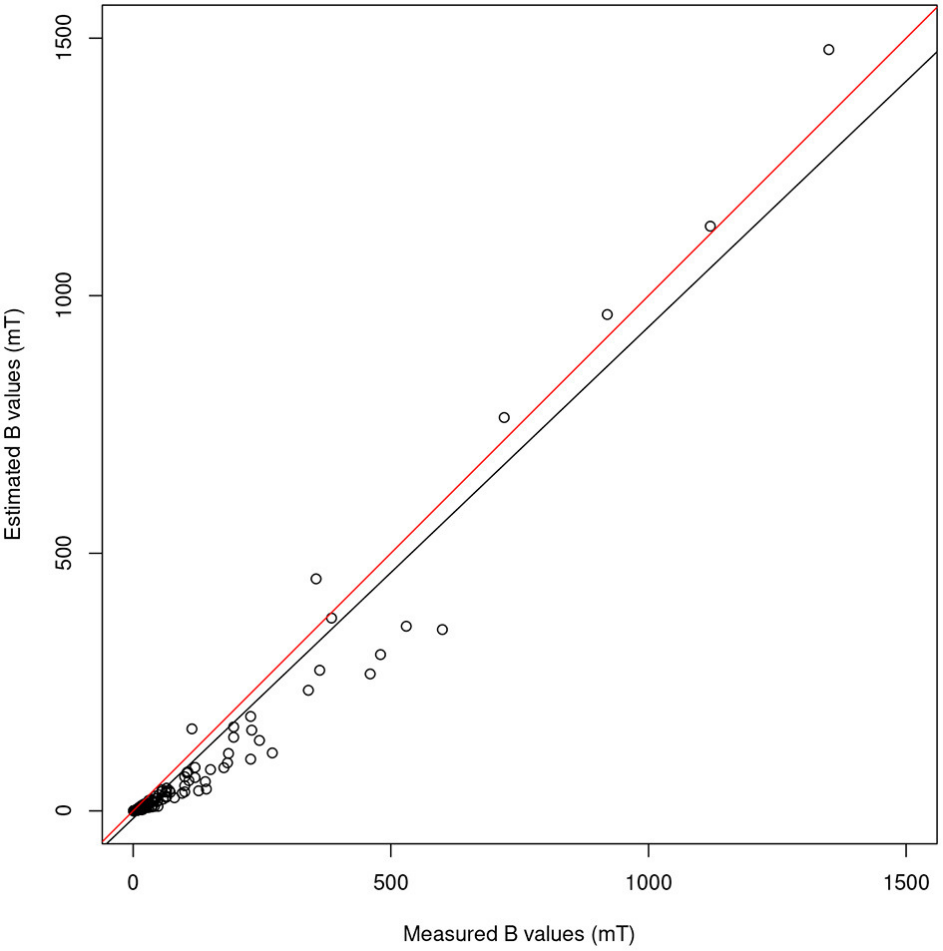
For the 3.0 T machine, scatter plot of all the 210 values of *B* estimated by the model proposed in Gurrera et al. (2019) vs. the raw measures. The black straight line shows the best fit, while the other represents “perfect” modeling, i.e., *y* = *x*.

Once conformed to the measured values, the dipole model in Gurrera et al. (2019) provides, in the case of the 3.0 T machine here analyzed, the response shown in Figure 5, displaying the scatter plot of all the estimated *B* values vs. the raw measures. Also displayed in the figure are two almost coincident straight lines, the black one being the best fit line, the other representing “perfect” modeling, i.e., *y* = *x*. As a result, despite the inherent heteroscedasticity, adherence to the model as measured by the Pearson's correlation coefficient is 0.97 (as it was in the case of the 1.5 T machines analyzed in Gurrera et al., 2019). The estimated values for the two unknowns Δ*x* and Δ*y* (Gurrera et al., 2019) are 69 and 8 cm, respectively.

### 3.3. Assessing the Whole-Body Exposure

**Figure 6:**
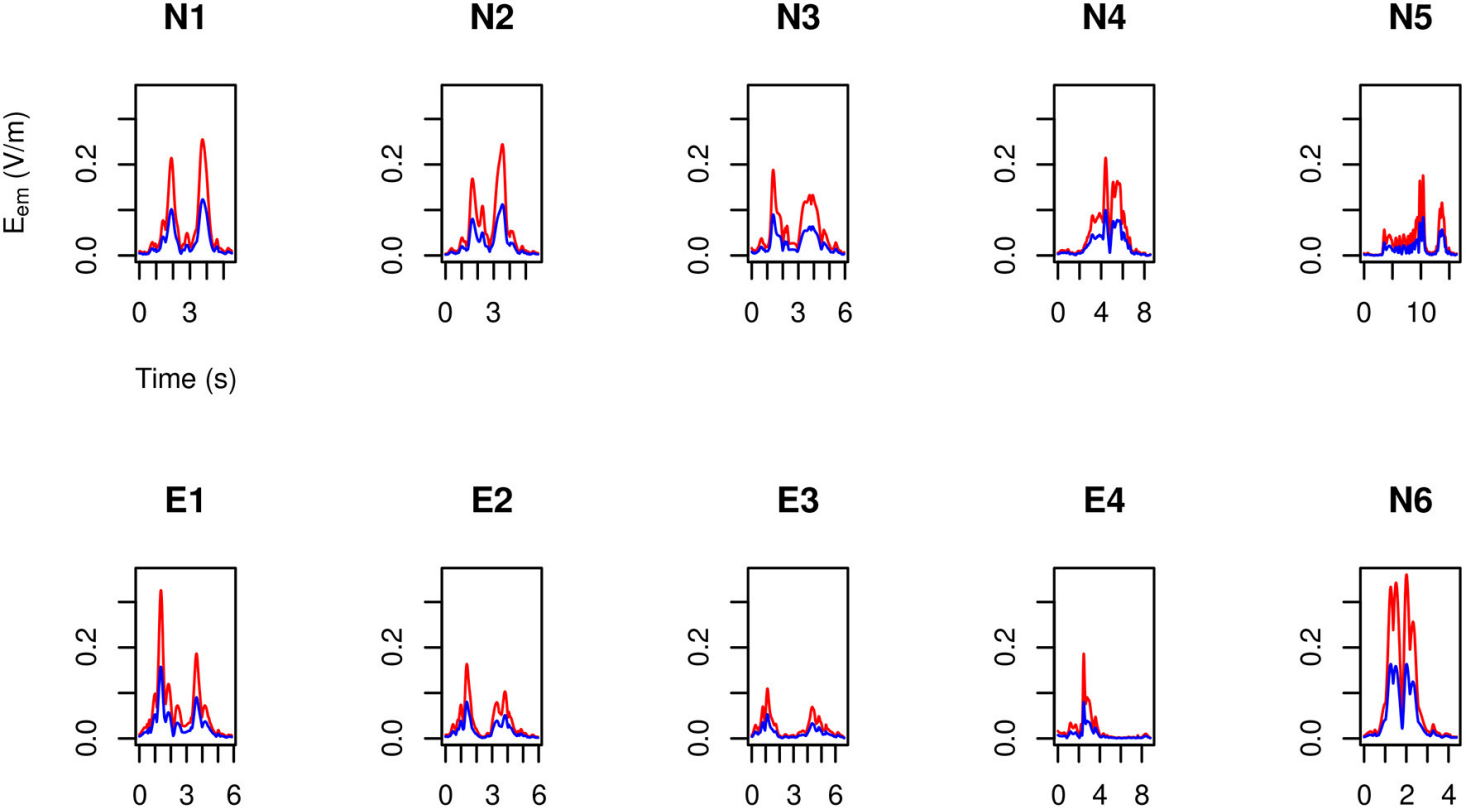
For MRIR1 (Table 1), body peak value of the motion induced electromotive field *E*_em_ during the execution of each of the recurrent selected tasks (Table 2). Particularly, the red line refers to the 3.0 T machine analyzed in this work, while the blue line refers to the 1.5 T Machine A analyzed in Gurrera et al. (2019).

**Figure 7:**
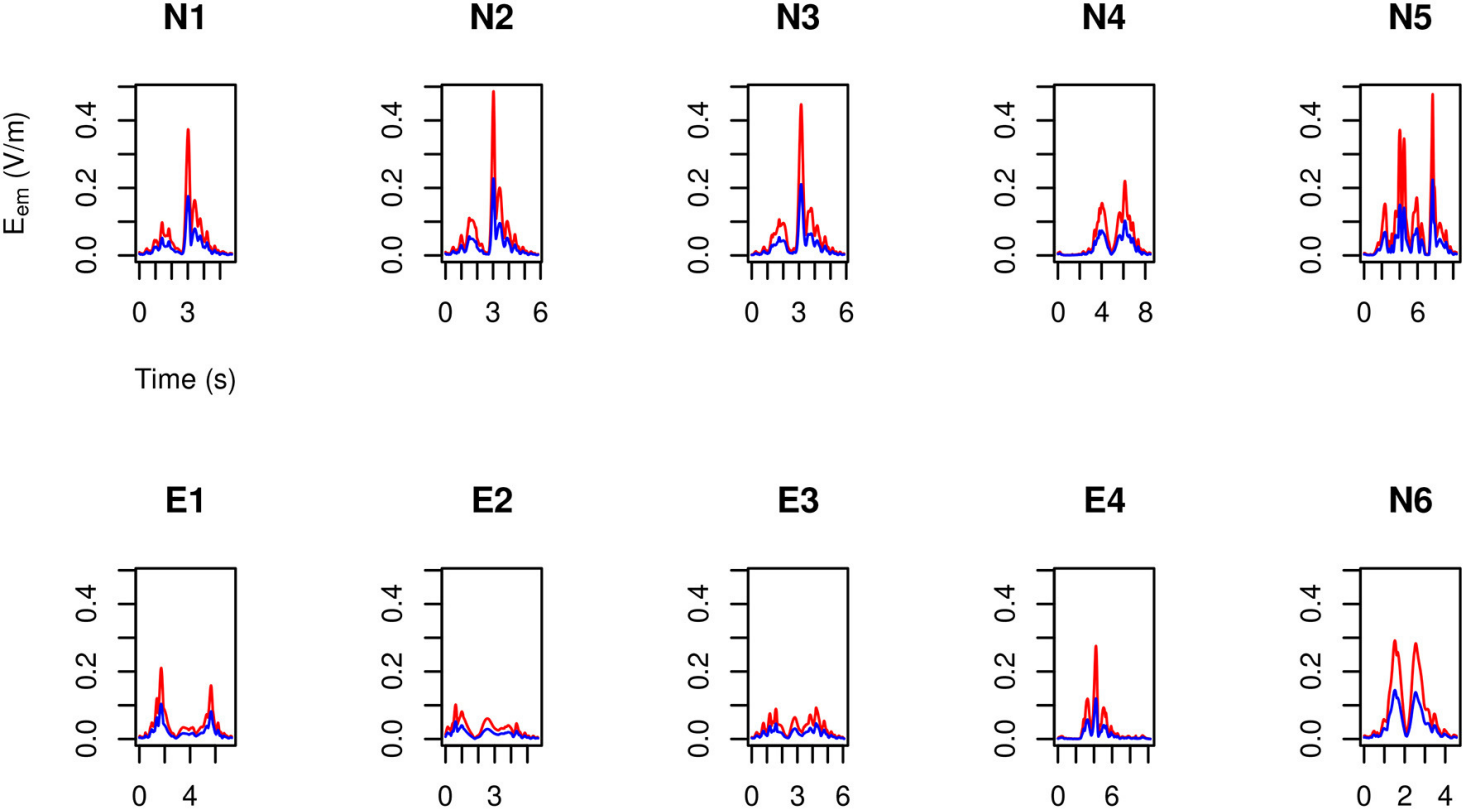
As in Figure 6, but for MRIR2.

**Figure 8:**
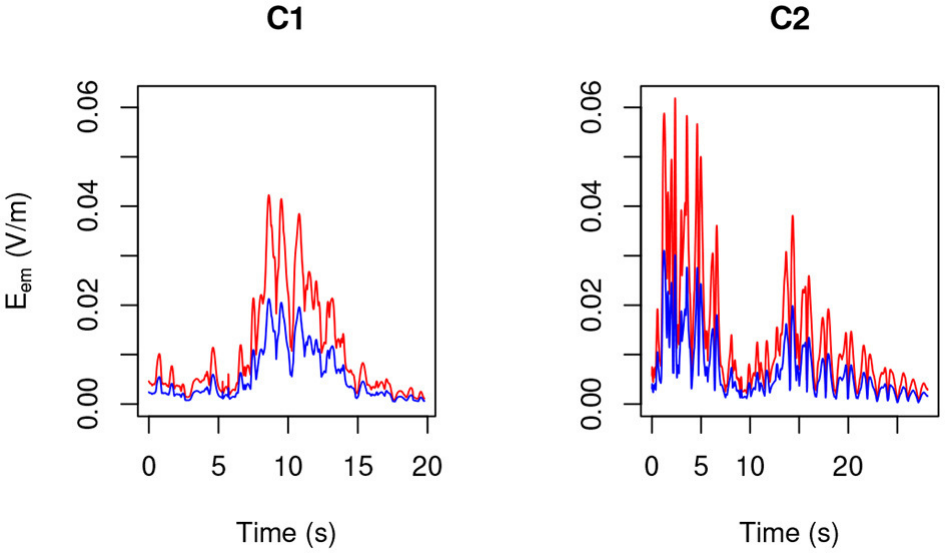
As in Figure 6, but for MRIC.

By making use of the position and velocity map of each of the 23 tracked points in the body of the three volunteers (Table 3) and of the $\vec{B}$ map provided by the model, in Figures 6–8 the peak value $\underset{i=1,\ldots ,23}{\max}|{\vec{v}}_{i}(t)\times \vec{B}|$ is shown during the (second) execution of each task. Results are given for the 3.0 T machine analyzed in this work, hereafter Machine 3.0, and for the 1.5 T Machine A analyzed in Gurrera et al. (2019).

For each operator and for each task, exposure results far below the 1.1 V m-1 ELV prescribed in European Union (2013) to account for possible health effects.

### 3.4. Assessing the Head Exposure

**Figure 9:**
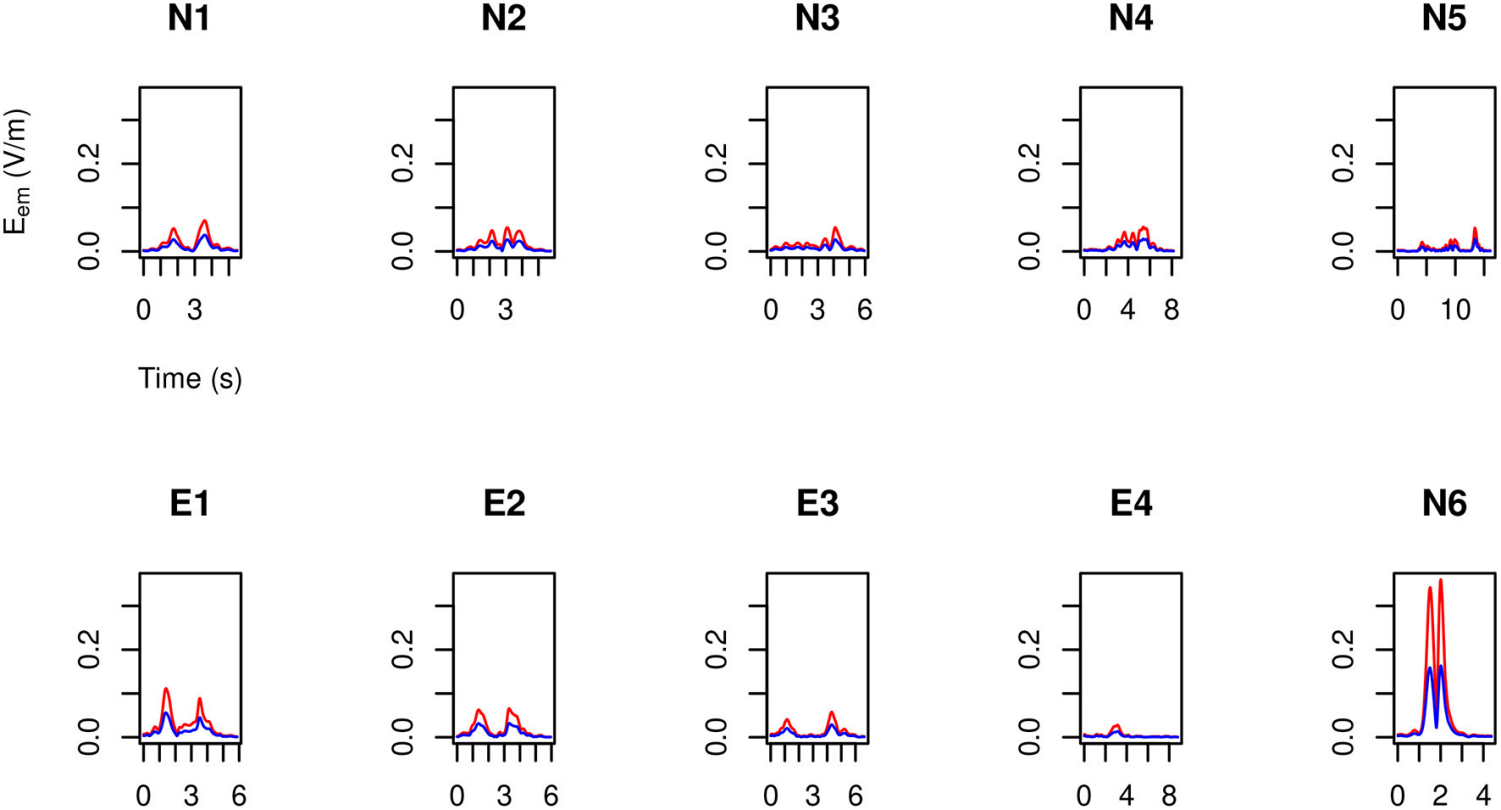
For MRIR1 (Table 1), head peak value of the motion induced electromotive field *E*_em_ during the execution of each of the recurrent selected tasks (Table 2). Particularly, the red line refers to the 3.0 T machine analyzed in this work, while the blue line refers to the 1.5 T Machine A analyzed in Gurrera et al. (2019).

**Figure 10:**
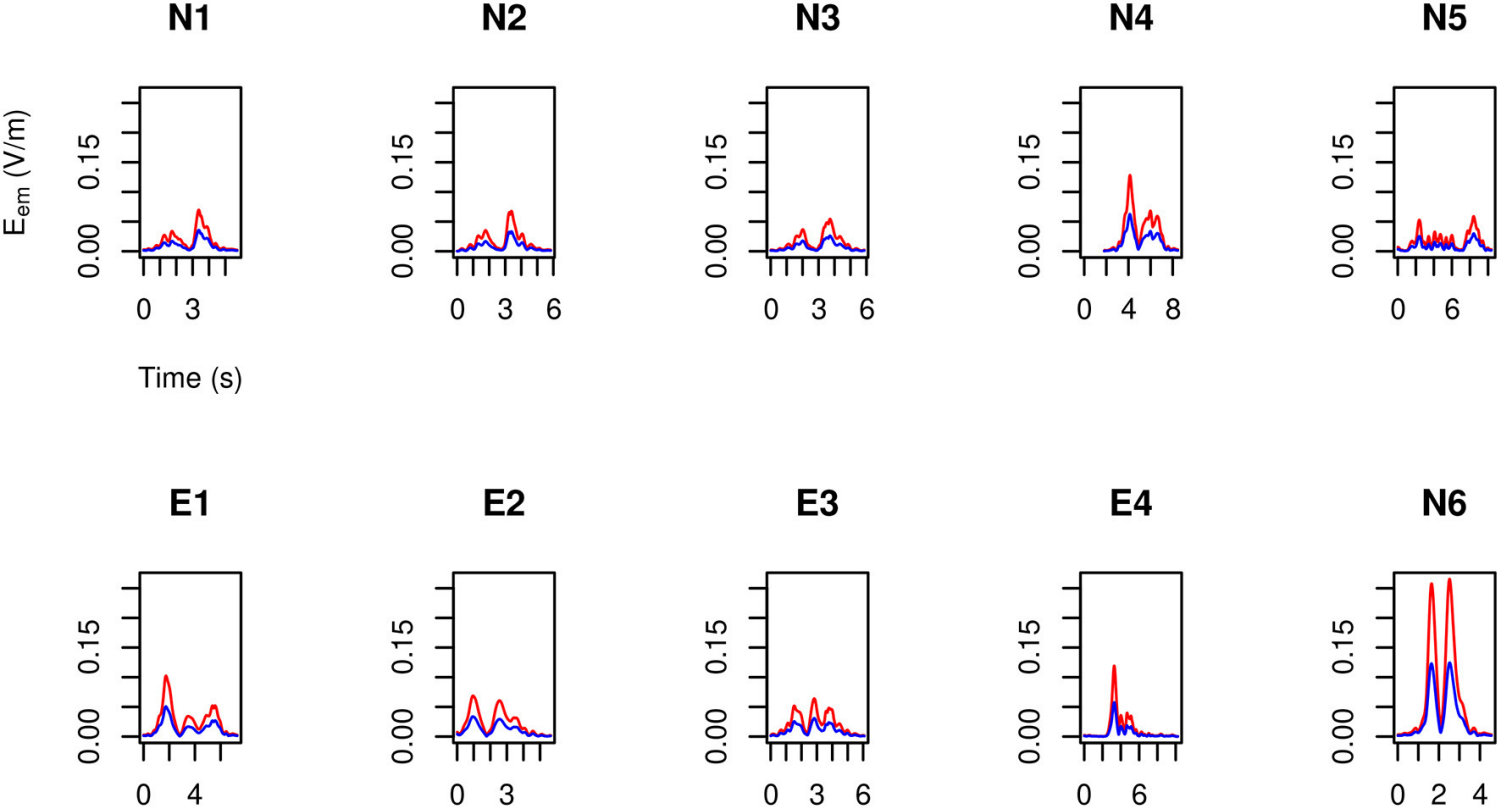
As in Figure 9, but for MRIR2.

**Figure 11:**
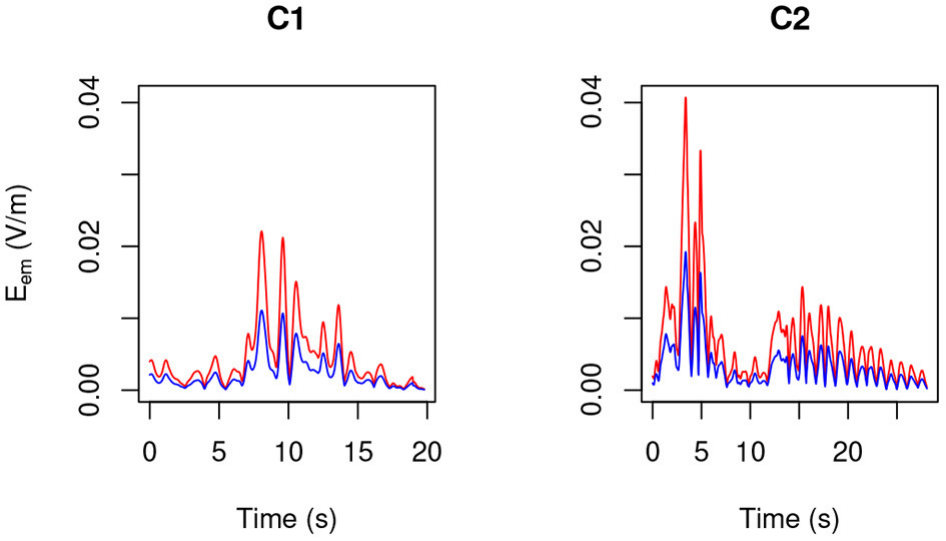
As in Figure 9, but for MRIC.

By making use of the position and velocity map of each of the five tracked points in the head of the three volunteers (Table 3) and of the $\vec{B}$ map provided by the model, in Figures 9–11 the peak value $\underset{i=1,\ldots ,23}{\max}|{\vec{v}}_{i}(t)\times \vec{B}|$ is shown during the (second) execution of each task.

**Figure 12:**
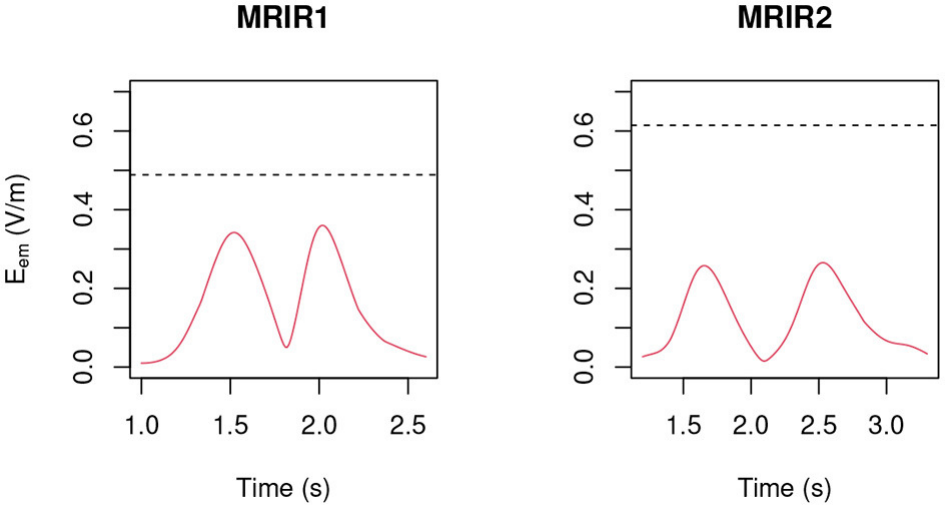
Induced pulse during “object recovering” (N6) for the two radiographers in the case of Machine 3.0, along with the proper ELV (dashed black line) adjusted by the motion-related frequency.

Only “object recovering” (N6) deserves being considered in detail and therefore in Figure 12 the induced pulse for MRIR1 and MRIR2 is shown in the case of Machine 3.0, along with the proper ELV adjusted by the motion-related frequency [1.43 and 1.14 Hz, respectively, as estimated by the spectral centroid of the corresponding periodogram (Massar et al., 2011)].

For each operator and for each task, exposure did not exceed the ELV prescribed in European Union (2013) to account for possible sensory effects, i.e., 0.7/f V m-1, where *f* is the motion-related frequency.

## 4. Discussion

In view of the obtained results, a limited risk margin (to exceed the ELV for the sensory effects) appeared only in the case of a 3.0 T machine. This raises questions about the opportunity of using lighter approaches. In fact, the “practical” reference levels in International Commission on Non-Ionizing Radiation Protection (2014) “for determining compliance with the basic restrictions for the induced internal electric field,” along with a practical but severe approximation of it (Equation 3), have proved to lead to alarming conclusions (inter alii, Acri et al., 2018 and Hartwig et al., 2019), while the estimation of the proper motion induced field introduced in Gurrera et al. (2019), once reliable measures of the components of the involved velocities and of the stray magnetic field are obtained, is definitely not less practical.

Here, in order to obtain realistic positions and velocities, a state-of-the-art HMA was used, able to track single reflective markers and relevant centroids with an accuracy smaller than 1 mm, and applied to three real MRI operators whose specific training and daily experience may be expected to replicate usual activities though in a simulated environment. Moreover, in order to span different typologies of workers, a man and a woman of very different height were recruited from a number of available radiographers. In this study, the “skin motion artifact,” due to the undesired displacements between the external markers on the skin and the underlying bone (Leardini et al., 2005), is not expected to affect the final results significantly, particularly the centroids, calculated from the external skin markers, and thus overall less sensible to this source of error.

In order to obtain the stray magnetic field, the simple dipole model already proposed in Gurrera et al. (2019) was used, which—once properly conformed to accurate static measures—has proved to yield reliable estimates of the three components of $\vec{B}$ also for a 3.0 T machine.

Of course, different scenarios other than those depicted here may occur in an MRI room and other different MRI facilities should be analyzed. The obtained results appear robust and suggest that, even in the case of a 3.0 T machine, a controlled behavior in the close proximity of the magnet might easily prevent electric pulses beyond the thresholds prescribed in the Directive 2013/35/EU. In any case, the present work represents an original cross-disciplinary interaction between physical models and human motion analysis, made nowadays more necessary for the complexities implied in these approaches, and casts light on the current lack of general consensus (Stam and Yamaguchi-Sekino, 2018). In fact, this lack appears to be due to three main reasons: (i) the ICNIRP has missed the fundamental role of the Lorentz' force; (ii) a lack of specialized studies dealing with the motion analysis of MRI operators; (iii) a lack of standardized MRI personnel training throughout the European Union. Particularly, the last point appears to be fundamental since, in view of the obtained results, it would provide safe working conditions at least up to 3.0 T machines for all MRI operators. Of course, it should account for each possible task and for different statures.

## 5. Conclusions

The present study developed a method for introducing real human motion data into an established model now able to represent the induced electric field in the human body in the vicinity of an MRI scanner. This model, once applied to the two machines here analyzed—1.5 T one, 3.0 T the other—has resulted in a final positive compliance statement: both the health and sensory effects ELVs prescribed in the Directive 2013/35/EU are not exceeded. According to the present results, the introduction of specialized training protocols for MRI personnel throughout the European Union would prevent any possible risk to exceed the thresholds prescribed by the Directive currently in force, at least up to 3.0 T machines.

## Data Availability Statement

The raw data supporting the conclusions of this article will be made available by the authors, without undue reservation.

## Ethics Statement

Ethical review and approval was not required for the study on human participants in accordance with the local legislation and institutional requirements. The patients/participants provided their written informed consent to participate in this study. Written informed consent was obtained from the individual(s) for the publication of any potentially identifiable images or data included in this article.

## Author Contributions

VC, DG, and AL conceived the study and were in charge of overall direction and planning. KG and BA conceived an *ad hoc* precision carpet to map the static magnetic field and DG realized it. CR fabricated an *ad hoc* precision support for the magnetometer. DG carried out the static magnetic field measurements with support from VC, KG, BA, GI, GA, and GV. AL and MO carried out the human movement analysis with support from SD and DG. DG and AL wrote the manuscript. DG designed the theoretical model, carried out its implementation, and analyzed the data. MM provided an in-depth reading and analysis of the manuscript. All authors provided critical feedback and helped shape the research and analysis.

## Conflict of Interest

The authors declare that the research was conducted in the absence of any commercial or financial relationships that could be construed as a potential conflict of interest.
